# Cytotoxic n-Hexane Fraction of the Egyptian *Pteris vittata* Functions as Anti-breast Cancer Through Coordinated Actions on Apoptotic and Autophagic Pathways

**DOI:** 10.1007/s12010-023-04464-3

**Published:** 2023-03-23

**Authors:** Khalid M. Mohany, Abo Bakr Abdel Shakour, Sara Ibrahim Mohamed, Randa Samir Hanna, Ahmed Y. Nassar

**Affiliations:** 1 Department of Medical Biochemistry and Molecular Biology, Faculty of Medicine, Assiut University, Assiut, 71515 Egypt; 2 Laboratory of Molecular and Cell Biology, Department of Zoology, Faculty of Science, Assiut University, Assiut, 71515 Egypt; 3 Assiut University, Assiut, 71515 Egypt

**Keywords:** PV-n-hexane extract, MCF-7, Autophagy, Apoptosis, P53

## Abstract

We investigated the possible anticancer mechanisms of *Pteris vittata* [PV] n-hexane extract on MCF-7 [breast cancer cell line]. Cultured cell lines were treated with various concentrations of this extract ± Baf-A1 [autophagic inhibitor]. Cells’ viability, apoptotic markers [caspase-7, Bax, and Bcl-2], autophagic markers [light chain 3 [LC-3] and P62/SQSTM1]], and the tumor suppressor P53 and its mRNA were checked by their corresponding methods. Treated cell lines showed significant concentration and time-dependent reductions in cell viability in response to PV*-*n-hexane extract and also exhibited a concomitant induction of apoptosis [increased chromatin condensation, nuclear fragmentation, and pro-apoptotic Bax, and cleaved caspase-7 levels while decreased Bcl-2 levels] and autophagy [increased autophagosomes vacuoles, and LC3B II levels while decreased P62/SQSTM1 levels]. Moreover, PV*-*n-hexane extract-treated cells showed significant increases in the P53 and its mRNA levels. The addition of Baf-A1 reversed the PV*-*n-hexane extract autophagic effects and increased apoptotic cell percentage with a much increase in the cleaved caspase-7 and P53 protein and its mRNA levels. We concluded that the PV*-*n-hexane extract exhibits cytotoxic effects on the MCF-7 cell line with significant reductions in cell viability and concomitant autophagy and apoptosis induction. Inhibition of autophagy in the PV-treated MCF-7 cells enhances apoptosis via a p35-dependent pathway.

## Introduction

Breast cancer is the most frequent tumor affecting women in the USA and worldwide [1]. Despite advances in therapy during the last few decades, more than 40,000 women still pass away every year in the USA, and one in three patients globally because of this type of cancer [2]. Surgery is the treatment of choice for breast cancer, but in many circumstances, to improve the outcome, it is followed or preceded by medication [adjuvant and neoadjuvant therapies, respectively] [3].

Several authentic anticancer drugs extracted from terrestrial plants [used as traditional medicine] have exhibited cytotoxic activities in either vitro or in vivo studies [4]. *Pteris vittata* (PV), a herbaceous fern, usually grows in tropical and subtropical areas and is known as an arsenic hyperaccumulator [5]. The n-hexane extracted fraction of PV has been found to contain a cytotoxic oxygenated acyclic sesquiterpenoid. This PV n-hexane extract is successfully used in traditional medicine as a therapeutic agent [6]. Also, it has shown anticancer activity on various cancerous human cell lines including the Michigan Cancer Foundation-7 (MCF-7; human breast cancer cell line) [7, 8].

Most cytotoxic agents act through the modulation of cellular autophagy and/or apoptosis to destroy cancer cells. This modulation can exert therapeutic features through the promotion of the two predicted events considered in cancer therapy, i.e., cell death and/or survival [9]. In response to many cellular stresses including cytotoxic chemicals in natural products, autophagy is mostly achieved as a pro-survival mechanistic pathway in both normal and tumorous cells [10]. Indeed, both apoptosis and autophagy have shown controversial roles in the process of tumorigenesis that may emphasize the harmony between both conditions [11–13].

The current work aimed to test the effect of the PV n-hexane extract on the cell viability of the MCF-7 cell line. Also, it investigated the impact of this extract on the expression of pro-apoptotic markers (caspase-7 and Bax), anti-apoptotic maker (Bcl-2), and the tumor suppressor P53 protein and its mRNA. Moreover, it examined the effect of this extract on the autophagy process by investigating its action on the microtubule-associated protein-1 light chain 3 (LC-3) and sequestome-1 (ubiquitin-binding protein p 62 or P62/SQSTM1).

## Materials and Methods

### Materials

To achieve the study’s aim, a pure sample of this extract was obtained from Gaafar’s associate [7]. Dulbecco’s modified eagle medium (DMEM) with L-glutamine, fetal bovine serum (FBS), and antibiotics (10.000 units/ml for both penicillin and streptomycin) were bought from Gibco (Invitrogen, CA. USA). Bafilomycin A1 (Baf-A1 (0.1 µM), a known inhibitor for the latter stages of autophagy), monodansyl cadaverine (MDC), and anti-sequestome-1 rabbit polyclonal IgG antibody were purchased from Sigma Aldrich. Anti bax (E63) IgG rabbit monoclonal antibody, anti-Bcl-2 (E17) IgG rabbit monoclonal antibody, anti-caspase 7 IgG monoclonal rabbit antibody, and anti p53 IgG mouse monoclonal antibody were bought from Abcam. Enhanced chemiluminescent (ECL) substrate was obtained from Thermo Scientific (Rockford, USA). Rabbit polyclonal anti-IgG antibody was bought from Invitrogen. Rabbit polyclonal IgG and rabbit anti-β actin IgG antibodies were bought from Abcam. Rabbit anti-LC3B IgG antibody was from Cell Signaling Technology. Horseradish peroxidase (HRP) conjugated goat anti-rabbit and rabbit anti-mouse IgG were from Santa Cruz Biotechnology. Other chemicals, if not otherwise mentioned, were purchased from trusted local suppliers.

### Ethics

The study was revised and approved by the local ethics committee, Faculty of Medicine, Assiut University, and given IRB#17,300,800.

### Cell Culture

The human MCF-7 cells, M.D. Anderson – Metastatic Breast 231 cell line (MD-A-MB-231), pro-monocytic, human myeloid leukemia cell line (U937), human embryonic kidney 293 cells (HEK 293), and hepatoma G2 and a human liver cancer immortal cell line (Hep G2) were obtained from VACSERA–Cell Culture Unit (Dokky, Giza, Egypt). All procedures were accomplished using mycoplasma-free cells. Cells were cultivated in DMEM with L-glutamine augmented with 10% (v/v) heat-inactivated FBS, 100 units/ml penicillin, and 100 µg/ml streptomycin at 37 °C in a humidified atmosphere of 95% air and 5% CO_2_.

#### ***A 3-(4,5-Dimethylthiazol-2-yl)-2,5-Diphenyl-2H-Tetrazolium Bromide (MTT) Assay ***[14]

In 96-well plates, cells (2 × 10^3^ cells/well) were placed for 24 h, and then the media was replaced by one containing various concentrations of the PV n-hexane extract alone (25 µg/ml, 50 µg/ml, and 100 µg/ml dissolved in dimethyl sulfoxide) or combined with the autophagy inhibitor, Baf-A1. To test the viability of different tested cell lines, the medium was changed to fresh medium augmented with 2 mg/ml MTT and then kept for 3 h at 37 °C after 24 h, 48 h, and 72 h. Any formed formazan crystals were dissolved by adding 100 µl of10% sodium dodecyl sulfate. The control cells were treated with the media only instead of the PV n-hexane extract. The absorbance was measured at 570 nm with 630 nm as a reference wavelength. Data were presented as the average percentage of viable cells compared to control according to the following equation:

$$growth inhibition=\left(100-\frac{\mathrm{absorption of the PV n}-\mathrm{hexane extract}-\mathrm{treated cells }}{\mathrm{absorption of the control cells}}\right)\times 100$$

The median inhibition concentration (IC_50_) of cells was determined.

### Monodansyl Cadaverine [MDC] Staining

To label the autophagic vacuoles (autophagosomes), the cells were incubated for 10 min with MDC (0.05 mM) in phosphate buffer solution (PBS) at 37 °C. After washing 3 times with PBS, cells were fixed on glass slides and examined by a fluorescence microscope (Nikon, Germany). Images were taken, and the MDC puncta were counted using ImageJ software (particle count). The number of Puncta of randomly 30 cells was counted, and then the main puncta per 1 cell was calculated.

### Western Blotting Analysis

After cell treatments, cells lysis was done with ice-cold radioimmunoprecipitation assay (RIPA) buffer (50 mM Tris–Cl (pH 7.6), 5 mM ethylenediamine tetra-acetic acid (EDTA), 150 mM NaCl, 0.5% NP-40, and 0.5% Triton-X-100) containing 1 μg/ml leupeptin and aprotinin and 0.5 mM phenylmethylsulfonyl fluoride (PMSF). After centrifugation of the samples at 2500 rpm for 10 min, the supernatants were aspirated. Protein concentration was measured by Bradford assay. Aliquots of proteins were separated by SDS–PAGE using 10% gels or 12% in the case of LC3 blotting. After transfer, nitrocellulose membranes were blocked with 2% bovine serum albumin (BSA) and probed with primary antibodies overnight at 4 °C. Membranes were then incubated with HRP-conjugated secondary antibodies (1:10,000) for 1 h at room temperature. Detection was performed using the ECL substrate. The densitometry of immunoreactive bands was estimated by ImageJ freeware.

### 4′,6-Diamidino-2-Phenylindole (DAPI) Staining

Cells fixation was done in 4% paraformaldehyde for 30 min and then washed 3 times with PBS before being stained in the dark with 300 nM DAPI for 5 min at room temperature. After the removal of DAPI, the cells were washed again 3 times and then examined by a fluorescence microscope (Nikon, Germany).

### Flow Cytometry

The effect of treatment with PV n-hexane extract on the MCF-7 cell viability is tested by flow cytometry. The MCF-7 was treated for 24 h with 0.25 µg/ml and 0.50 µg/ml of PV n-hexane extract alone or mixed with Baf-A1.

### Reverse Transcription Polymerase Chain Reaction [RT-PCR] for the P53

Total RNA was isolated using the RNeasy Mini Kit. cDNA was synthesized by Affinity Script Multiple Temperature cDNA Synthesis Kit according to the manufacturer’s protocol. The obtained cDNA was amplified using the following primers for human p53 (primer Bank ID: 40804465c2) forward primer: 5'-ACTTGTCGCTCTTGAAGCTAC-3' and reverse primer: 5'-GATGCGGAGAATCTTTGGAACA-3' and for the equal loading reference (GAPDH) (primer Bank ID: 378404907c3) forward primer: 5'-CTGGGCTACACTGAGCACC-3' and reverse primer: 5'-AAGTGGTCGTTGAGGGCAATG-3'. The conditions of PCR amplification for P53 were 94 ℃ initial denaturation for 5 min, followed by 30 cycles each of 94 ℃ for 30 s, 57 ℃ for 30 s, and 72 ℃ for 90 s), followed by a final extension at 72 ℃ for 5 min. Aliquots from the obtained amplicons were subjected to 2% agarose electrophoresis, and band density was estimated by Image J freeware.

### Statistics

Quantitative results were presented as means ± SD and analyzed by one-way analysis of variance, followed by Newman-Keuls post-test. Results were considered statistically significant at *p* < 0.05.

## Results

### Effect of PV n-Hexane Extract Treatment on Cell Viability

**Fig. 1:**
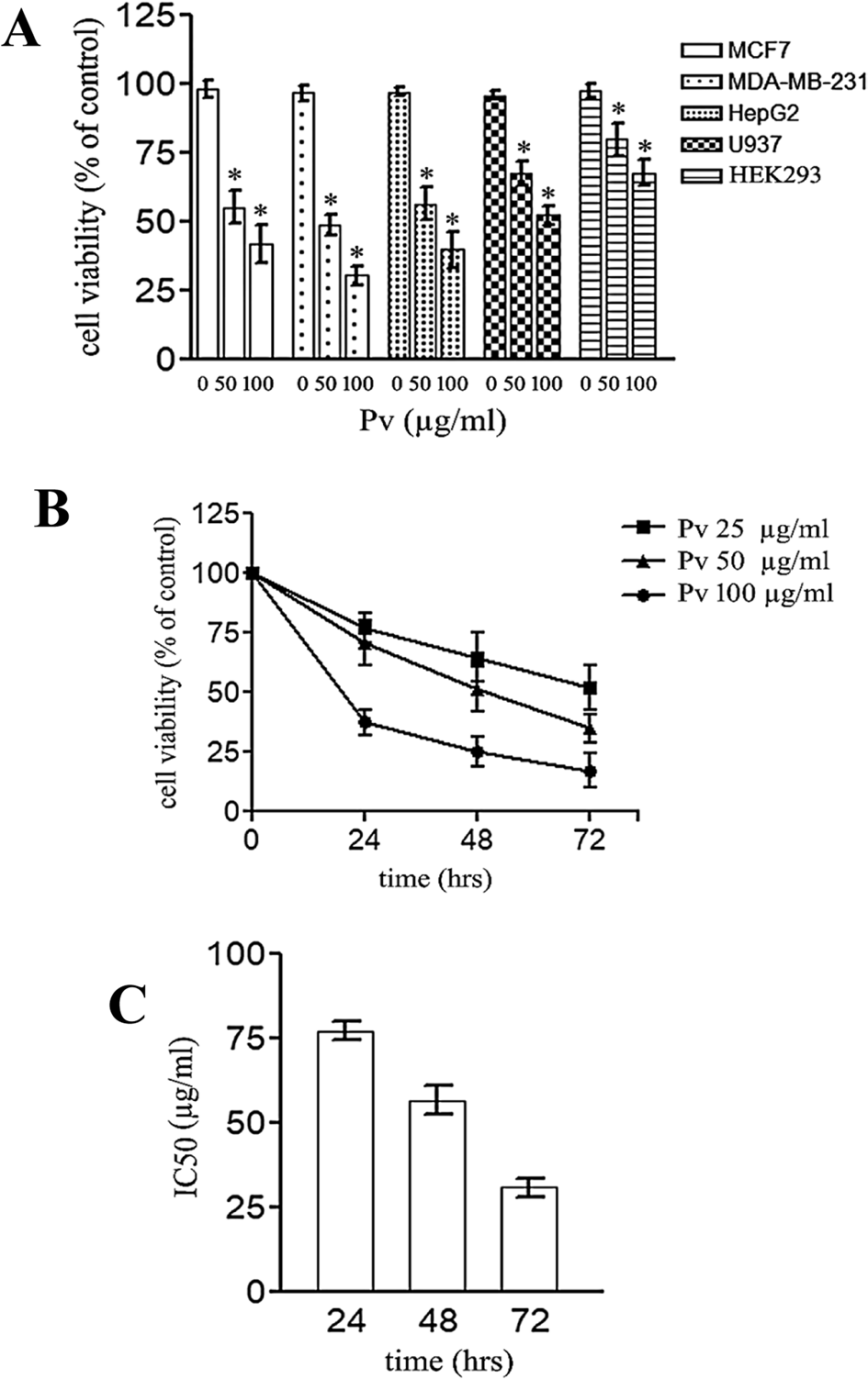
MTT assay for cell viability after treatment with the PV n-hexane extract: MTT cell viability assay for different cell lines after treatment with the mentioned concentrations of the PV n-hexane extract for 24 h [**A**]. MTT assay for MCF7 cell line treated with the mentioned concentrations of the PV n-hexane extract for 24 h, 48 h, and 72 h [**B**]. IC_50_ of the PV n-hexane extract for 24 h, 48 h, and 72 h [**C**]. Data are means of three independent experiments ± SD [*p < 0.05]

The PV n-hexane extract showed significant concentration-dependent reductions in the cell viability of the cancerous cell lines of MCF-7, MDAMB-231, Hep G2, U937, and HEK-293 assayed levels by the MTT method (Fig. 1A). Among the tested cell lines, HEk293 cells showed the least while the MDA-MB-231 cells showed the greatest reduction in the cell viability in response to the PV n-hexane extract. Also, The MCF-7 cells showed time and concentration-dependent response behavior toward the tested PV n-hexane extract (Fig. 1B). The estimated IC50 of PV n-hexane extract on MCF-7 cells was 74.5 µg/ml, 53.2 µg/ml, and 29.5 µg/ml for 24 h, 48 h, and 72 h, respectively (Fig. 1C).

### Induction of Apoptosis and Autophagy by the PV n-Hexane Extract

**Fig. 2:**
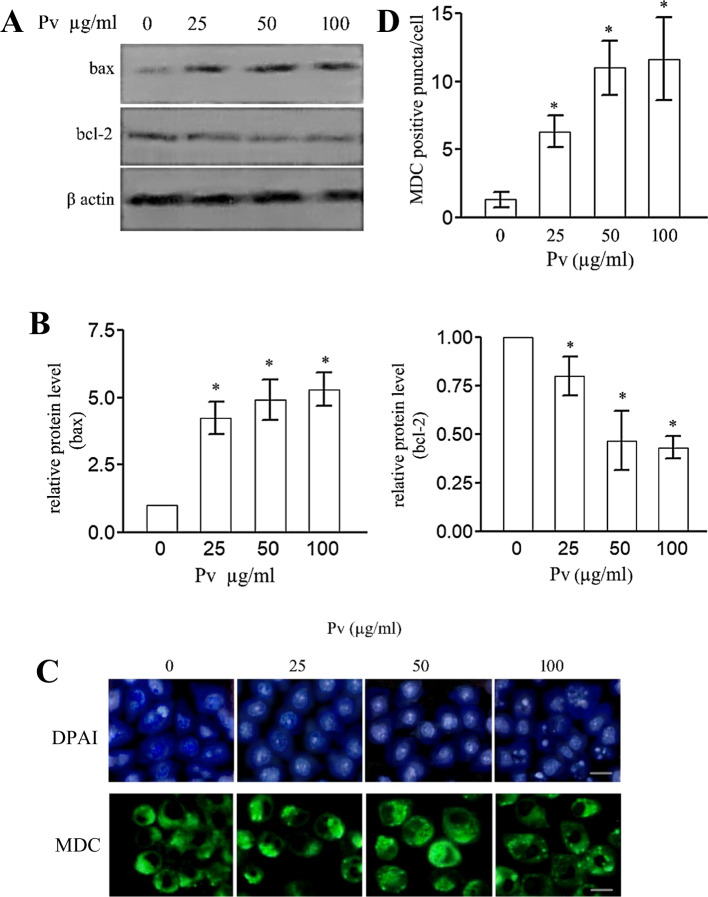
The PV n-hexane extract induces apoptosis and autophagy. Cells were treated or not with the PV n-hexane extract [25 µg/ml, 50 µg/ml, and 100 µg/ml] for 24 h. Western blotting analysis of the level of Bax and BcL-2 was carried out; the shown image is a representative one, and actin was used as an equal loading reference [**A**]. The relative protein levels were quantified densitometrically [**B**]. Cells were subjected to DAPI staining for the detection of nuclear morphology and MDC staining for autophagy vacuoles detection [**C**]. MDC puncta/cell was calculated as the mean of puncta, *n* = 30 [**D**]. Data are means of three independent experiments ± SD [*p < 0.05]. Scale bar = 5 µm

The current work found a significant elevation of the Bax levels and reduction in the Bcl-2 levels in the PV n-hexane extract-treated MCF-7 cell lines compared to the control (Fig. 2A). Such a modulation by PV n-hexane extract showed a concentration-dependent pattern (Fig. 2B). For DAPI staining, the nuclear chromatin was homogenous in untreated cells, while condensed and fragmented in the PV n-hexane extract-treated cells indicating apoptosis that also showed a concentration-dependent pattern (Fig. 2C – upper panel).

The MDC staining displayed a rise in the number of MDC-stained puncta (i.e., an increase in the autophagosomes and autolysosomes) in PV n-hexane extract-treated cell lines, and this increase was also concentration-dependent after only 6 h of incubation (Fig. 2C – lower panel and Fig. 2D).

### Inhibition of the PV n-Hexane Extract-induced Autophagy Enhances the MCF-7 Apoptosis

**Fig. 3:**
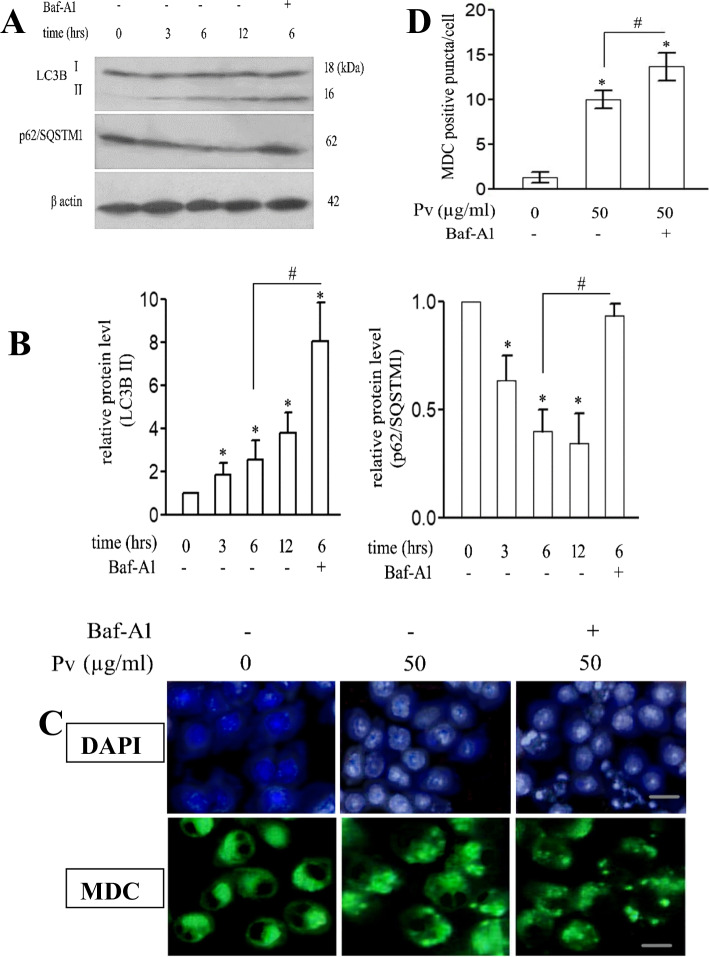
Inhibition of PV n-hexane extract-induced autophagy enhances its apoptotic activity. Cells were treated or not with PV n-hexane extract [50 μg/ml] and Baf-A1 (0.1 μM) for 3 h, 6 h, and 12 h. The levels of LC3 B and p62/SQSTM1 were detected by western blot; the shown image is a representative one; actin was used as an equal loading reference [**A**]. The relative protein levels were quantified densitometrically [**B**]. Cells were treated or not with the PV n-hexane extract [50 μg/ml] and Baf-A1 for 1 h and then subjected to DAPI staining for detection of nuclear morphology and MDC staining for autophagy vacuoles detection [**C**]. MDC puncta/cell was calculated as the mean of puncta, *n* = 30 [**D**]. Data are means of three independent experiments ± SD [*p < 0.05]. Scale bar = 5 μm

The current study found a gradual increase in the LC3B level with a concurrent decline in the P62/SQSTM1 levels in the PV n-hexane extract-treated MCF-7 cells until 12 h of treatment. The Baf-A1 addition reversed this effect within 6 h (Fig. 3A, B) and increased the chromatin condensation, nuclear fragmentation, and the MDC-stained puncta (Fig. 3C,D).

**Fig. 4:**
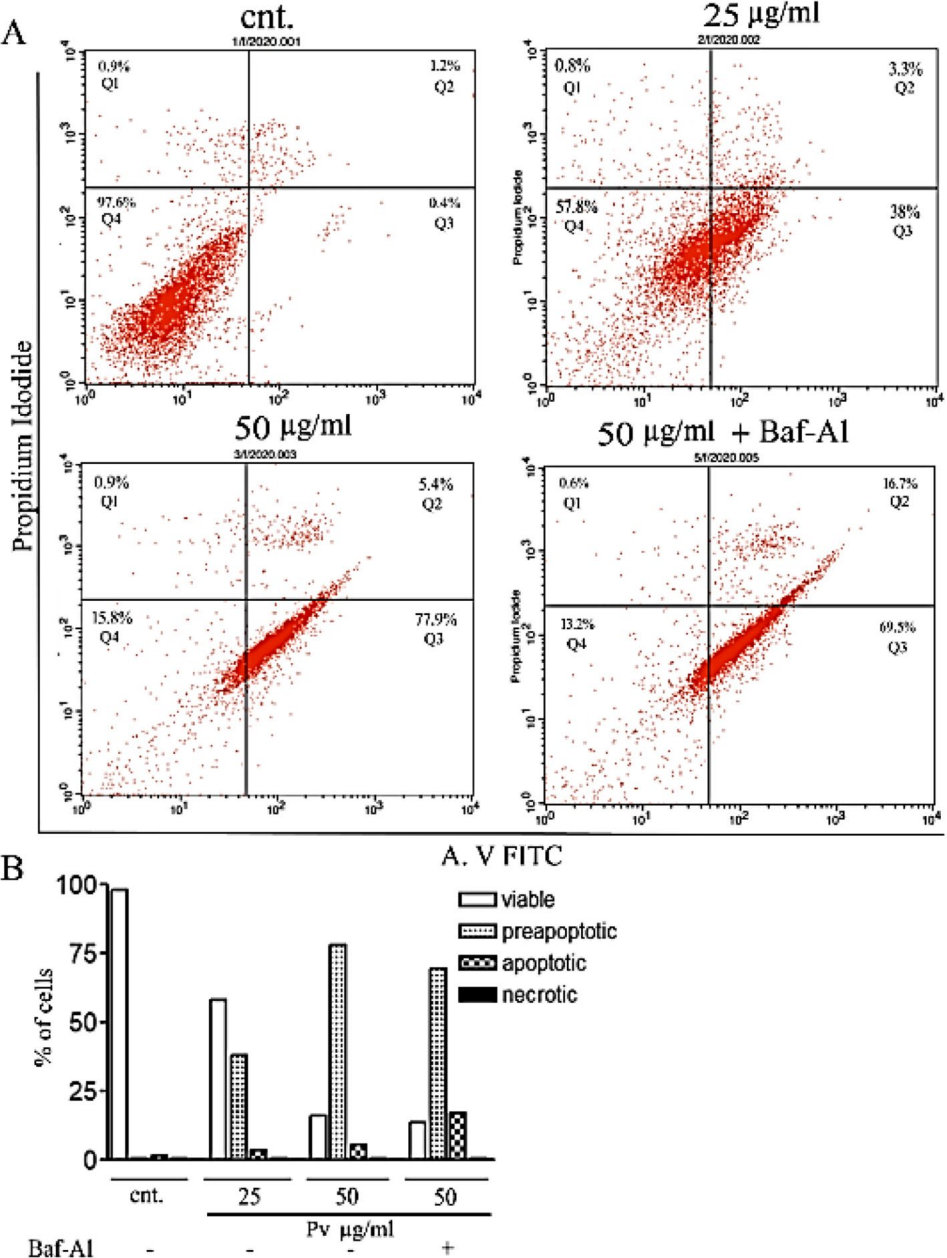
Effect of PV n-hexane extract treatment on cell viability measured by flow cytometry. Representation flow cytometry results for MCF-7 cells treated with PV n-hexane extract 0 µg/ml, 25 µg/ml, 50 µg/ml, and 50 µg/ml PV n-hexane extract plus Baf-A1 illustrating the cell staining with propidium iodide [PI] and [fluorescein isothiocyanate] FITC annexin V **[A].** Q1: necrotic cells; Q2: late apoptotic cells; Q3: early apoptotic cells; Q4: living cells. Percentage of living, early apoptotic, late apoptotic, and necrotic cells [**B**]. Data are presented as means ± SD of three independent experiments. *p < 0.05, as compared with the control group

Moreover, by flow cytometry, the PV n-hexane extract-treated MCF-7 cells showed an increase in the percentage of the pre-apoptotic and apoptotic cells. The addition of Baf-A1 (0.1 µM) to these cells increased the percentage of the apoptotic cells at the expense of the pre-apoptotic cells (Fig. 4A, B).

### Caspase-7 and P53 Protein and mRNA in the PV n-Hexane Extract-treated MCF-7 Cell Line

**Fig. 5:**
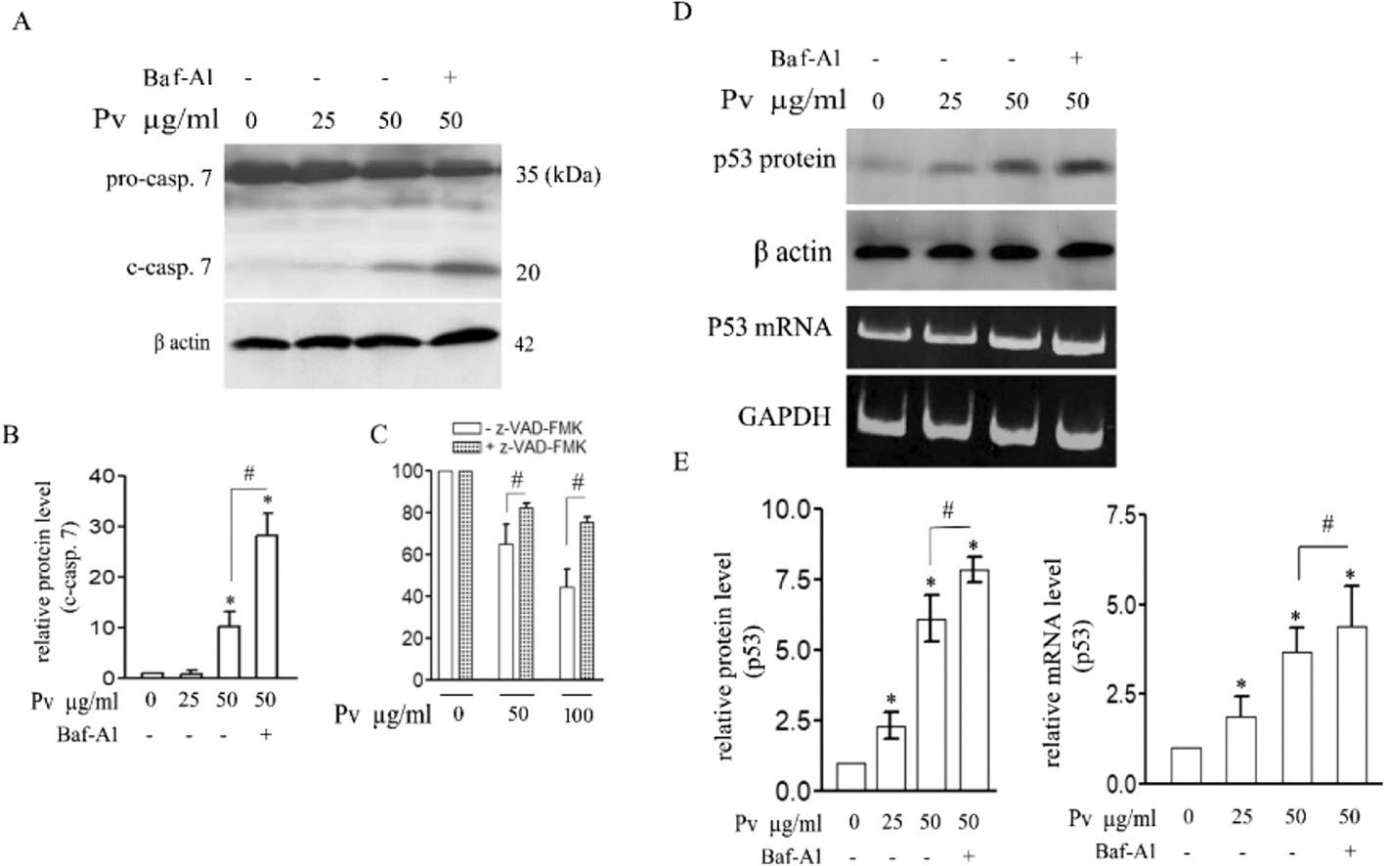
Role of caspase 7 and p53 in PV n-hexane induced apoptosis and autophagy. Representative western blot image showing the level of procaspase 7 [pro-casp. 7] and cleaved caspase 7 [c-casp. 7] after 24 h of PV n-hexane treatment with the mentioned concentrations; autophagy was inhibited by Baf-A1 cotreatment; actin was used as an equal loading reference [**A**]. The relative protein level of c-casp. 7 was quantified densitometrically [**B**]. MTT assay result for cell viability after exposure of cells to 0 µg/ml, 50 µg/ml, and 100 µg/ml of PV n-hexane with or without z-VAD-FMK [caspases inhibitor] [**C**]. Representative western blot image showing the level of p53 protein level after cell treatment as described; actin was used as an equal loading reference [**D**]. Representative image of RT-PCR for semi-quantification of mRNA level of P53 gene after treatment of cells as described; GAPDH mRNA level was used as equal loading reference [**D**]. Data are presented as mean ± SD of three independent experiments. * and # *p* < 0.05, as compared with the control group

The current experiment found a gradual PV n-hexane extract concentration-dependent increase in the levels of the cleaved caspase-7 (C-Cap. 7) and P53 protein and mRNA in the MCF-7 cells. These increases were much high up on the Baf-A1 treatment (Fig. 5A,B,D,E).

When the caspases’ activity was inhibited by the Z-VAD-FMK in the PV n-hexane extract-treated MCF-7 cells, the viability of these cells increased (Fig. 5C).

## Discussion

Natural products and traditional medicine have been very much considered in the field of cancer therapies because of their biological safeness with potential and efficient therapeutic effects. More than 53% of anticancer drugs recorded in medicine are unchanged natural products of mixture components, botanic drugs, and byproducts of natural products [15]. In this regard, the chemical composition and pharmacological activity of n-hexane extracted fraction from the pantropical genus *Pteris vittata* have been executed [8]. This extract contains alkane hydrocarbons, oxygenated sesquiterpenoid, doxan, corticosterone, and other organic components. It was proven to be cytotoxic and has anticancer activities against various human tumor cell lines including MCF-7 [7].

In this study, the effect of a pure sample of PV n-hexane extract on cell viability, apoptotic, and autophagic response in different human cell lines, especially breast cancer MCF-7, has been investigated. Our findings have shown significant concentration and time-dependent reductions in the viability of different PV n-hexane extract-treated cell lines tested by the MTT method. The reductions were more highly manifested in the rapidly dividing cell lines than in the normally divided control ones. These results emphasize the previously reported cytotoxicity of the PV n-hexane extract [7].

In general, chemotherapy and cytotoxic agents aim to destroy the tumor cells, and this requires a strict harmony between autophagy and apoptosis; otherwise, the tumor cells will survive [9]. Both features are crucial for maintaining cellular homeostasis through their nonstop crosstalk to regulate and coordinate cell survival/breakdown [16]. Our findings showed a concomitant induction of both apoptosis and autophagy in the PV n-hexane extract-treated MCF-7 cell line.

Apoptosis occurs as a physiological response to many types of extracellular and intracellular insults. Any dysregulation in this process leads to the development of many diseases including tumorigenesis [17, 18]. The key to apoptosis is the activation of the caspases [cysteine proteases] that start a cascade of consequences, resulting in the degradation of the cellular macromolecules [19]. Apoptosis is finely tuned through the controlled balance between the pro-apoptotic proteins [Bax] and anti-apoptotic proteins [Bcl-2] [20].

In the present experimental study, the PV n-hexane extract-treated MCF-7 cell line exhibited concentration-dependent elevations in the pro-apoptotic Bax concentrations and reductions in those of the anti-apoptotic Bcl-2 with signs of nuclear chromatin condensation and fragmentation [by DAPI stain].

Balanced autophagy is a cell-survival mechanism that involves the destruction and/or regeneration of outdated or injured macromolecules. Autophagy may suppress or enhance tumor growth [13]. Many inhibitors or activators for autophagy have been listed as organic chemical extracts from natural products that can promote successful anticancer effects [21]. During the autophagy process, the development of the intracellular autophagosomes is important to deliver the damaged and undesirable macromolecules [e.g., proteins and toxins] to the lysosomes [form autolysosomes] for their destruction and recycling. By doing this, autophagy protects the cells from the hazards of these unwanted macromolecules [22]. Dysregulation of the autophagy process due to genetic or metabolic causes may affect the process of tumorigenesis [23].

The progress of autophagosomes is controlled by many proteins including Beclin-1 and autophagy-related proteins [ATGs]. These proteins recruit the LC3 to expand and elongate the phagophore [24]. The process of recruitment involves the conversion of pro-LC3 to LC3 I [cytosolic] and then to LC3 II [lipidated membrane-bound]. The latter helps the autophagosomes to bind the unwanted macromolecules and autophagosome-lysosome fusion [25]. Also, the protein p62/SQSTM1 acts as an autophagosome receptor that binds both the ubiquitinated cargo and LC3B [26]. It is degraded during this course of action, so a reduction in the p62/SQSTM1 concentrations occurs with the increase in autophagy activity [27].

Mild cellular insults activate autophagy, but when it is severe, apoptosis occurs. When the autophagy process is initiated, apoptosis is usually repressed. Also, once apoptosis starts, the activated caspases cleave the proteins involved in the autophagic process, and autophagy stops [19, 28]. There are proteins involved in the regulation of both apoptosis and autophagy. These proteins include the Bcl-2 subfamilies, Bak, Bax, and Bik, and beclin-1 [16, 19, 29, 30].

Our study of LC3B and P62/SQSTM1 autophagy markers by western blotting showed a gradual increase in LC3B II levels [LC3IB was converted into its matured lipidated form LC3IIB] while a gradual decline in the P62/SQSTM1 levels in the PV n-hexane-treated MCF-7 cell lines until 12 h which indicated the induction of autophagy. This effect was reversed by the concurrent treatment with Baf-A1 for 6 h. In such conditions, the two LC3B forms were mostly equalized, and the sequestrating adaptor protein receptor p62 was seemingly unchanged or affected.

On the examination of the effect of autophagy inhibition on the apoptotic cell death induced by PV n-hexane extract treatment, cells were treated with PV n-hexane extract [50 µg/ml] with or without Baf-A1 for 12 h, and then the cells were stained with DAPI and MDC staining for detection of apoptosis and autophagy, respectively. The autophagy inhibitor Baf-A1 led to accumulations of more autophagy vacuoles as expected compared with cells treated to the cells treated only with the PV n-hexane extract. Interestingly, the number of condensed chromatin and fragmented nuclei also was elevated in the Baf-A1 cotreatment, which indicated that inhibition of autophagy by the PV n-hexane extract enhanced the apoptotic activity. These findings went with Mariño et al. [2014], who reported an enhancement in autophagy on the elimination of the apoptotic Bax and Bak or by adding a caspase inhibitor [19].

A more detailed study concerning the effect of PV n-hexane extract-induced autophagy on cell viability was carried out by flow cytometry. It seemed that after 24 h treatment with 25 µg/ml and 50 µg/ml of PV n-hexane extract, the percentage of apoptotic and pre-apoptotic cells increased, but the sharpest increase was in pre-apoptotic cells. After inhibition of autophagy with Baf-A1, the percentage of apoptotic cells increased, but pre-apoptotic cells decreased compared to the PV n-hexane extract-only-treated cells.

In our experiment, we excluded the investigation of caspase 3 because it is mutated and functionless in MCF-7 [31]. So, we examined the role of caspase 7. It was obvious that caspase 7 is incorporated in PV n-hexane extract-induced apoptotic cell death. There was a gradual elevation of the level of the c-cap. 7 over increasing concentrations of PV n-hexane extract [0 µg/ml, 25 µg/ml, 50 µg/ml]. Upon inhibition of autophagy with Baf-A1, the level of caspase-7 increased compared to cells without Baf-A1. This confirmed the role of caspase 7 in apoptotic cell death induced by PV n-hexane extract and also confirmed the pro-survival role of autophagy induced by PV n-hexane extract. To investigate the incorporation of caspases as general in the apoptotic cell death induced by PV n-hexane extract, cells were treated with the general caspases’ inhibitor z-VAD-FMK, and then cell viability was assessed by MTT assay. Treatment with z-VAD-FMK significantly protected cells from the apoptotic effect of PV n-hexane extract at different concentrations. These results clarified the role of caspases in general in the apoptotic pathway induced by PV n-hexane extract.

The P53 [tumor suppressor] is one of the proteins that help autophagy-apoptosis harmony [16]. Its role in autophagy is contradicting. On the one hand, it induces autophagy-related genes such as ATGs, and on the other hand, it suppresses autophagy by inducing the degradation of autophagic inducer beclin-1 [32, 33]. This means autophagy and p53 are always in coordination in most signaling pathways. We decided to investigate if PV n-hexane extract-induced autophagy is p53-dependent. The levels of P53 protein and mRNA were estimated after exposure of cells to 0 µg/ml, 25 µg/ml, and 50 µg/ml of the PV n-hexane extract with or without autophagy inhibitor [Baf-A1]. The levels of p53 protein and mRNA upregulated gradually over increasing concentrations of PV n-hexane extract. The Baf-A1 cotreatment resulted in a significant increase in p53 protein and mRNA levels compared with cells treated with PV n-hexane extract only.

## Conclusion

The PV n-hexane extract has a cytotoxic effect on the MCF-7 cell lines that are associated with a significant reduction in the cell viability and concomitant induction of both autophagy and apoptosis. Inhibition of autophagy while treating the MCF-7 cells with PV n-hexane extract resulted in an enhancement of the apoptosis via a p35-dependent pathway. We recommend that testing the possible molecular mechanisms of cytotoxicity of individual components of the PV n-hexane extract should be taken into consideration in future research. Also, the use of an early-step autophagic inhibitor with the PV n-hexane extract instead of the Baf-A1 [late autophagic inhibitor] should be tested as we think it may give better results.

## Data Availability

Data are contained within the article.

## Abbreviations

**USA**: United States of America
**PV**: N-hexane of *Pteris vittate*
**MCF-7**: Michigan Cancer Foundation-7
**Caspase**: Cysteine aspartate protease
**Bcl-2**: B-cell lymphoma 2
**Bax**: Bcl-2-associated X protein
**P53**: Is a tumor suppressor protein 53-kilodalton protein
**LC3**: Microtubule-associated protein light chain 3
**SQSTM1/p62**: Sequestosome-1 or the ubiquitin-binding protein p62
**IRB**: Institutional Review Board
**DMEM**: Dulbecco’s modified eagle medium
**FBS**: Fetal bovine serum
**Baf-A1**: Bafilomycin A1
**MDC**: Monodansyl cadaverine
**IgG**: Immunoglobulin G
**ECL**: Enhanced chemiluminescence luminal
**HRP**: Horseradish peroxidase
**MDAMB-231**: M.D. Anderson – Metastatic Breast 231 cell line
**U937**: Human myeloid leukemia cell line
**HEK 293**: Human embryonic kidney 293 cells
**VACSERA**: Holding company for biological products of vaccines and sera
**MTT**: The 3-[4,5-dimethylthiazol-2-yl]-2,5-diphenyl-2H-tetrazolium bromide
**IC**_**50**_: Median inhibition concentration
**PBS**: Phosphate buffer saline
**RIPA**: Radio immunoprecipitation assay
**EDTA**: Ethylene diamine tetra-acetic acid
**BSA**: Bovine serum albumin
**PI**: Propidium iodide
**FITC**: Fluorescein isothiocyanate
**GAPDH**: Glyceraldehyde 3-phosphate dehydrogenase
**z-VAD-FMK**: Carbobenzoxy-valyl-alanyl-aspartyl-[O-methyl]-fluoromethyl ketone
**BIK**: BCL-2 interacting killer

## References

[1] Organization WH. A short guide to cancer screening: Increase effectiveness, maximize benefits and minimize harm. 2022.

[2] DeSantis CE, Bray F, Ferlay J, Lortet-Tieulent J, Anderson BO, Jemal A (2015). International variation in female breast cancer incidence and mortality ratesinternational variation in female breast cancer rates. Cancer Epidemiology, Biomarkers & Prevention.

[3] Fenton A, Downes N, Mendiola A, Cordova A, Lukity K, Imani J (2022). Multidisciplinary management of breast cancer and role of the patient navigator. Obstetrics and Gynecology Clinics.

[4] Fridlender M, Kapulnik Y, Koltai H (2015). Plant derived substances with anti-cancer activity: From folklore to practice. Frontiers in Plant Science.

[5] Xu W, Du Q, Yan S, Cao Y, Liu X, Guan D-X (2022). Geographical distribution of As-hyperaccumulator Pteris vittata in China: Environmental factors and climate changes. Science of the Total Environment.

[6] Xin G, Ye G, Li P, Tang W-J, Gao J-L, Zhao W-M (2008). Cytotoxic diterpenoids and sesquiterpenoids from *Pteris multifida*. Journal of Natural Products.

[7] Gaafar Alaa A, Ali Sami I, Faried Ahmed M, El-Hallouty SM (2018). An insight into chemical content, biological effect and morphological features of Pteris vittata L, rarely growing in Egypt. Research Journal of Chemistry and Environment.

[8] Hou M, Chen Y, Wang Y, Hao K (2021). Sesquiterpenoids and flavonoids from *Pteris multifida* Poir. Biochemical Systematics and Ecology.

[9] Hseu Y-C, Tsai T-J, Korivi M, Liu J-Y, Chen H-J, Lin C-M (2017). Antitumor properties of Coenzyme Q0 against human ovarian carcinoma cells via induction of ROS-mediated apoptosis and cytoprotective autophagy. Scientific Reports.

[10] Klionsky DJ, Abdel-Aziz AK, Abdelfatah S, Abdellatif M, Abdoli A, Abel S (2021). Guidelines for the use and interpretation of assays for monitoring autophagy 1. Autophagy.

[11] Tardáguila M, Mañes S (2013). CX3CL1 at the crossroad of EGF signals: Relevance for the progression of ERBB2+ breast carcinoma. Oncoimmunology.

[12] Arandjelovic S, Ravichandran KS (2015). Phagocytosis of apoptotic cells in homeostasis. Nature Immunology..

[13] Colhado Rodrigues BL, Lallo MA, Perez EC (2020). The controversial role of autophagy in tumor development: A systematic review. Immunological Investigations.

[14] Mosmann T (1983). Rapid colorimetric assay for cellular growth and survival: Application to proliferation and cytotoxicity assays. Journal of Immunological Methods..

[15] Newman DJ, Cragg GM (2020). Natural products as sources of new drugs over the nearly four decades from 01/1981 to 09/2019. Journal of Natural Products..

[16] Das S, Shukla N, Singh SS, Kushwaha S, Shrivastava R (2021). Mechanism of interaction between autophagy and apoptosis in cancer. Apoptosis.

[17] Feng S, Zha Z, Wang Z, Yang P, Wu J, Li X (2021). Anticancer activity of oleiferoside B involving autophagy and apoptosis through increasing ROS release in MCF-7 cells and SMMC-7721 cells. Natural Product Research.

[18] Singh R, Letai A, Sarosiek K (2019). Regulation of apoptosis in health and disease: The balancing act of BCL-2 family proteins. Nature Reviews Molecular Cell Biology..

[19] Mariño G, Niso-Santano M, Baehrecke EH, Kroemer G (2014). Self-consumption: The interplay of autophagy and apoptosis. Nature Reviews Molecular Cell Biology.

[20] D'Orsi B, Mateyka J, Prehn JH (2017). Control of mitochondrial physiology and cell death by the Bcl-2 family proteins Bax and Bok. Neurochemistry International..

[21] Al-Bari M, Alim A, Ito Y, Ahmed S, Radwan N, Ahmed HS (2021). Targeting autophagy with natural products as a potential therapeutic approach for cancer. International Journal of Molecular Sciences.

[22] Shaid S, Brandts C, Serve H, Dikic I (2013). Ubiquitination and selective autophagy. Cell Death & Differentiation..

[23] Vessoni A, Filippi-Chiela E, Menck CF, Lenz G (2013). Autophagy and genomic integrity. Cell Death & Differentiation.

[24] Mochida K, Oikawa Y, Kimura Y, Kirisako H, Hirano H, Ohsumi Y (2015). Receptor-mediated selective autophagy degrades the endoplasmic reticulum and the nucleus. Nature.

[25] Lőrincz P, Juhász G (2020). Autophagosome-lysosome fusion. Journal of Molecular Biology.

[26] Rogov V, Dötsch V, Johansen T, Kirkin V (2014). Interactions between autophagy receptors and ubiquitin-like proteins form the molecular basis for selective autophagy. Molecular Cell.

[27] Klionsky DJ, Abdel-Aziz AK, Abdelfatah S, Abdellatif M, Abdoli A, Abel S (2021). Guidelines for the use and interpretation of assays for monitoring autophagy. Autophagy.

[28] Maiuri MC, Zalckvar E, Kimchi A, Kroemer G (2007). Self-eating and self-killing: Crosstalk between autophagy and apoptosis. Nature Reviews Molecular Cell Biology.

[29] Germain M, Mathai JP, Shore GC (2002). BH-3-only BIK functions at the endoplasmic reticulum to stimulate cytochrome c release from mitochondria. Journal of Biological Chemistry.

[30] Kang R, Zeh HJ, Lotze MT, Tang D (2011). The Beclin 1 network regulates autophagy and apoptosis. Cell Death and Differentiation.

[31] Jänicke RU (2009). MCF-7 breast carcinoma cells do not express caspase-3. Breast Cancer Research and Treatment.

[32] Crighton D, Wilkinson S, O'Prey J, Syed N, Smith P, Harrison PR (2006). DRAM, a p53-induced modulator of autophagy, is critical for apoptosis. Cell.

[33] White E (2016). Autophagy and p53. Cold Spring Harbor Perspectives In Medicine.

